# A systematic review of RFID applications and diffusion: key areas and public policy issues

**DOI:** 10.1186/s40852-015-0010-z

**Published:** 2015-09-04

**Authors:** Kwangho Jung, Sabinne Lee

**Affiliations:** 1 Korea Institute of Public Affairs, Graduate School of Public Administration of Seoul National University, Seoul, Republic of Korea; 2 Graduate School of Public Administration of Seoul National University, Seoul, Republic of Korea

**Keywords:** RFID, Digital identification, Digital delivery, Smart tag, Contactless smart card

## Abstract

RFID applicants called as e-ID, smart tag, and contactless smart card are being applied to numerous areas in our daily life, including tracking manufactured goods, currency, and patients to payments systems. To review these various applications of RFID is important to exploring not only ongoing e-governance issues such as digital identification, delivery process, and governance but also business oriented application areas like supply chain. Through a systematic review methodology from 111 previous studies about RFID technology for public sector, we found six key areas of RFID applications: defense and security, identification, environmental applications, transportation, healthcare and welfare, and agriculture-livestock. We also suggest that the diffusion and applications of RFID can involve unexpected disadvantages including technological deficiency, uncertain benefits, dubious transparency, uncomfortable privacy issue, and unequal distribution of digital power and literacy. Further research on RFID impact includes not only various theoretical issues of but also legal and managerial problems. Rigorous research is required to explore what factors are critical to adopt and implement new RFID applications in terms of technology governance and digital literacy. Massive data driven research is also expected to identify RFID performance in government agencies and various industry sectors.

## Background

RFID technology has been widely implemented all over the world and its impact on our daily life is very diverse and massive (Li et al., 2006; Wyld, 2005). Those diverse areas of RFID application include logistical tracking, monitoring and maintenance of products, product safety and information, and payment process. Today many governments around the world in both developed^1^ and developing^2^ countries are trying to apply it for various areas from tracking manufactured goods, currency, and patients to securing sagety of payments systems. Massive RFID applications around all the industry sectors and countries are expected to generate a huge potential benefits for sustainable efficient energy infrastructure, transportation safety, and health care. Over the past 50 years, RFID technology went through innovations and progressions to become a more efficient and effective gadget for human beings as well as effective solutions of technical and organizational problems in various industry sectors. However, key issues of appropriate ICT technology, governing networks among RFID domains, standardization requirement, and privacy still remain unsolved^3^.

We review previous literature about RFID technology used in public sectors in order to identify what has been done and found to suggest policy implications and further research agenda. More specifically, we discuss four aspects regarding RFID research issues and policy implications. First, we examine various competing concepts of RFID use by governments all over the world. Second, we categorize numerous applications of RFID technology through analyzing previous literature. Third, we try to figure out technological issues and governance problems that RFID technology faces today. Last, we draw key public issues and suggest future research agenda.

## Methodology of the RFID literature review

### A brief history of RFID technology

RFID technology was emerged as Frederick Hertz found existence of radio frequency during his experiment in 1886 (Wyld, 2005) and developed for the purpose of defense during the Second World War^4^. During 1970s and 1980s, the RFID system attracted plenty of scholars and innovators, so efforts to register patents progressed (Takahashi, 2004). Researchers like Charles Walton had registered a patent to use RFID. In the 1980s, many US and European companies recognized the importance of developing RFID technology and started to manufacture RFID tags. Soon scholars at MIT University opened an Auto-ID center to promote the use and implementation of RFID technology. But most of the scholars report that the first commercialization of RFID technology was done by Wal-Mart as they launched RFID based material identifying system in 2005 (Shahram and Manish 2005). Wal-Mart is now tracking merchandise including food, apparels, and electronic items with RFID technology in their supply chain.^5^. RFID technology is a brand new policy tool that can ensure high transparency, efficiency and effectiveness not only in industrial areas but also in government service delivery. Table 1 describes a brief history of how RFID technology was developed and diffused.

**Table 1:** A brief history of RFID technology

Date	Event
1886	The idea of using Radio Frequency to reflect waves from objects was started from Frederick Hertz’s experiment.
1930–1940	American navy research laboratories developed a system known as IFF (Identify Friend or Foe).
1940–1950	The first application of RFID consisted of identifying allied or enemy planes during WW2 through the use of IFF system.
1973	Charles Walton, a former IBM researcher registered patent using RFID technology, a radio-operated door lock.
1980–1990	Many US and European companies started to manufacture RFID tags.
2003	The Auto-ID center for MIT became EPC global, an organization whose objective is to promote the use and adoption of RFID technology.
2005	Wal-Mart launched and RFID pilot.

### Research design for a systematic review

We searched online data base and expert based information to identify RFID publications between 2003 and 2015. We categorized RFID applications and analyzed issues and concerns that RFID faces today by systematically reviewing published literature. We have collected literature we use for systematic review from two different resources. First, most of the studies are found by searching the e-database. We could access electronic databases, such as Google Scholar, World Web of Science (WWS), Proquest Central, and Science Direct through Seoul National University’s main library homepage. We had set ‘RFID technology’, ‘RFID government,’ ‘RFID application, and ‘RFID issue’ as keywords for searching literature. We found most of the research through this method of searching. The second method we used for collecting data was having discussions with experts. To do this, we first made a list of experts who specialize in IT technology, Science technology, and public administration. Five experts agreed to help us and recommended some research papers that were known for their fluent flow of logic and plentiful contents. We chose relevant research papers from among experts’ recommendations. In sum we had used previous literature collected from two methods we discussed above, searching e-database and asking experts, as our resource of searching.

**Fig. 1:**
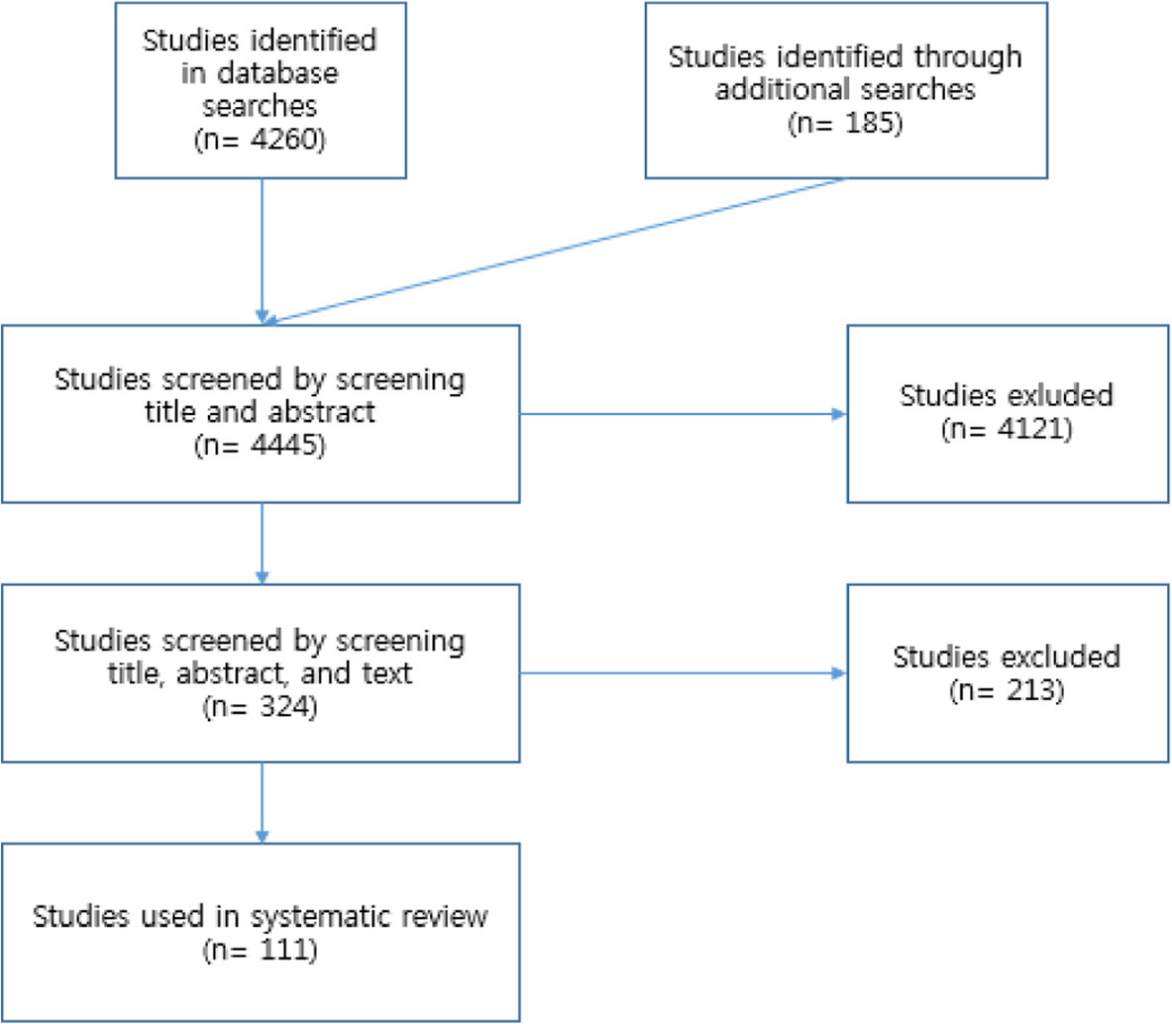
Analytical Frame

[Figure 1] shows analytical frame that we use for this study. We have determined the literature for systematic review according to three stages shown on the flow chart. First, the original total number of studies we have found from the e-database was 4260. Also 185 research papers were found from experts’ recommendations and previous public papers. A total of 4,445 studies were chosen through the first stage. Second, we excluded 4,121 following general eligibility criteria by screening title and abstract. More specifically, we excluded RFID studies only with one of the following criteria: 1) studies focusing on private sector; 2) studies without considering how public sector implemented RFID technology; 3) studies that did not discuss any social scientific implications; and 4) studies that only deal with RFID technology from pure scientific and engineering points of view. In sum, we included only 324 papers that discussed RFID issues and their implications in public sector. Third, we removed further 213 studies too much focusing on private sector or RFID technology itself, rather on its applications in our society and social scientific implications. Finally, 111 articles were chosen for our systematic review.

**Fig. 2:**
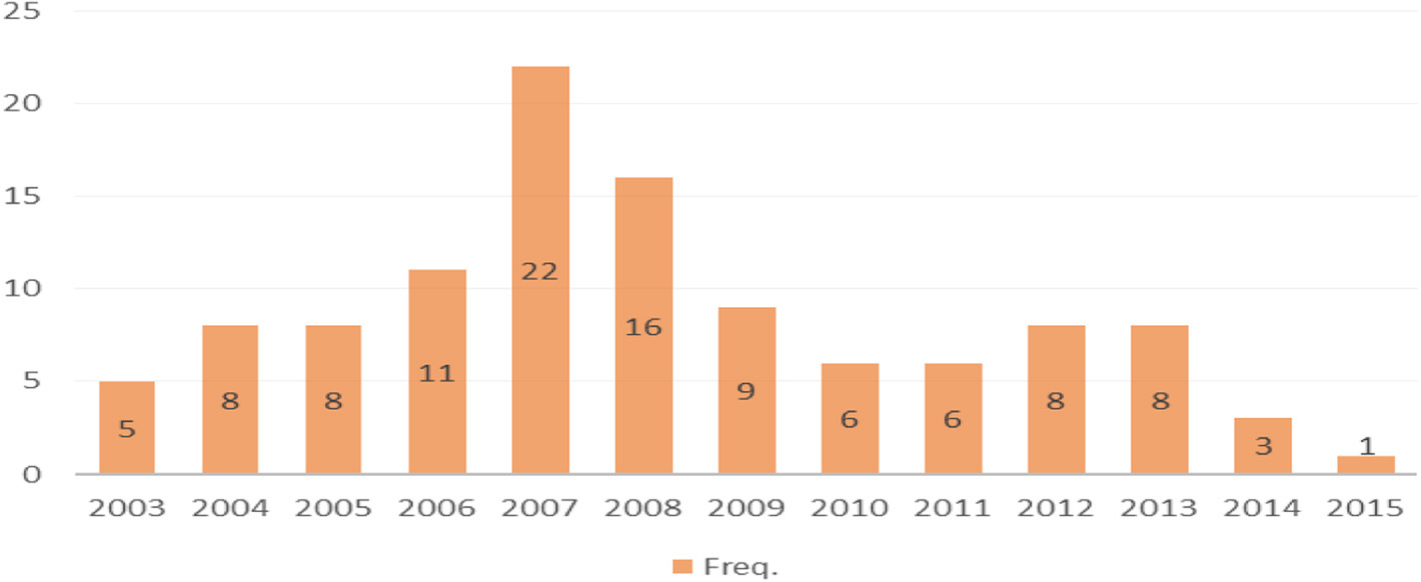
Descriptive Statistics of Literatures by year

[Figure 2] below showed descriptive statistics of collected literatures by published year. It shows 22 studies were published in 2007 among 111 literatures. As we already described above in history of RFID section, the popularization and commercialization of RFID technology was started in 2005 with Wal-Mart’s adoption. It seems that after Wal-Mart’s innovative footsteps hit the world, many scholars were started to recognize the potential of new technology and tried to understand and develop RFID technology. Besides some governments from all over the world implemented new way of public service delivery using RFID technology. Consequently, 49 literatures were publishedbetween 2006 and 2008 and it forms almost 45 % of our collected studies

**Fig. 3:**
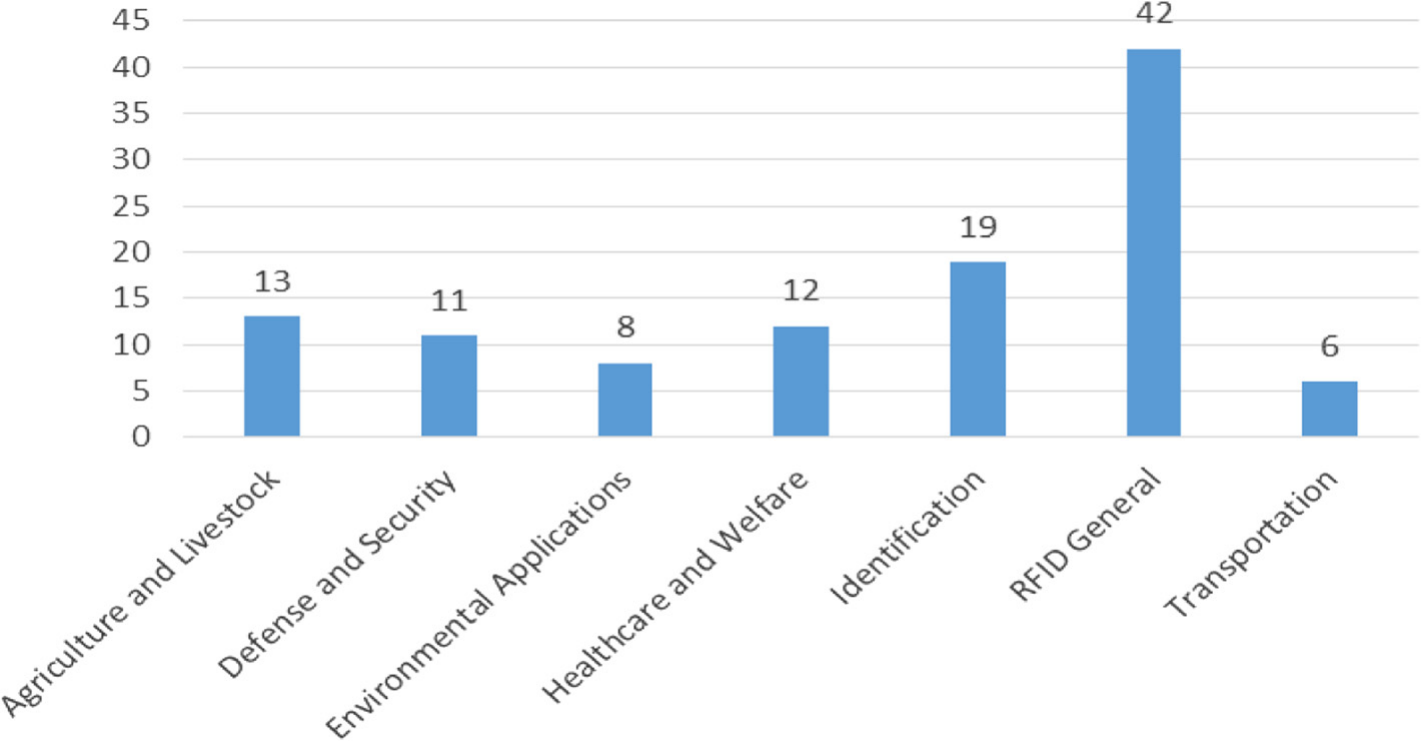
Descriptive Statistics of Literatures by applications

For this study, we categorized governments’ way of using RFID technology in 6 areas; Agriculture and Livestock, Defense and Security, Environmental Applications, Healthcare and Welfare, Identification, and Transportation. [Figure 3] shows descriptive statistics of collected literatures categorized by applications. We categorized studies that did not focus on specific sector and analyze and introduce RFID technology from the general perspectives as ‘RFID general’. ‘RFID general’ studies usually deal with various ways of using RFID technology in diverse sectors simultaneously. As we can see from [Fig. 3], RFID general area had 42 papers. That means still lots of RFID studies could not be fully specialized and remained in status of generally introducing RFID technology. Identification sector scores secondly highest number of published literatures among areas. This result seems natural because e-ID card or e-Passport have most powerful force that can hurt privacy, one of the most serious and notorious issues that RFID technology face today

## Key applied areas of RFID

### Defense and security

As we show in [Table 1], the history of RFID technology was started from the need for ensuring national security. Almost 60 years have passed since US army developed RFID based identification system to identify allies and enemies, RFID technology is still used for protecting people. For instance, Weinstein (2005) and Konsysnki & Smith (2003) reported how the US Army and Navy implement RFID technology in cargo containers to identify materials. The US Army and Navy implement RFID not only to identify US troops’ own weapons and containers but also to identify enemies in battle (Tien 2004)^6^. RFID systems are also important in terms of airport and port security. After the 9/11 terror attack on the United States, President George W. Bush let all the airport and port in US adopt identification systems based on RFID technology to protect its nation from additional terrorist attacks (Werb and Sereiko 2002)^7^. In 2012, the Taiwanese government decided to implement an RFID based e-Seal system to increase security and efficiency (Tsai and Huang 2012). In addition, RFID technology can be used effectively in prison management^8^ and child protection. In some countries like Japan and Republic of Korea, the RFID tag is implemented in child protection monitoring (Table 2).^9^

**Table 2:** RFID application in Defense and Security

Application	Author	Year	Case	Country
Army and Navy	Weinstein	2005	US Navy embedded RFID into cargo container	US
	Konsynski and Smith	2003	US Army track containers of material	US
	Tien	2004	US Army using RFID for tracking its army in Iraq	US
	Anon	2002	US Army piloted 4 projects using RFID	US
Airport and port Security	Werb and Sereiko	2002	2 RFID programs to prevent terrorist’s attack	US
	Smith and Konsynski Zhang	2003 2013	New York city government project using RFID, *CargoMate*	New York City, US
	Tsai and Huang	2012	Kaohsiung port adopt RFID system for port security	Taiwan
Prison management And Child protection	Kim	2008	Calpatria prison issued RFID embedded bracelet to its inmates	US
	Nicholas	2008	LA County prison management	US
	Ema and Fujigaki	2011	Let parents know the exact time of a child’s arrival and departure time	Japan
	Yonhap news	2013	Child monitoring through RFID tag in beach	ROK

### Identification

Electronic passports like ‘e-passports’ were adopted electrically after the 9/11 attack. After the terrible tragedy broke heart of United States, the American government became aware of the importance of checking VISAs and passports correctly. The US Department of State soon let people who wanted to enter US to use RFID tag embedded electronic passports instead of traditional barcode based passports^10^. The European Union also endorsed the inclusion of biological information in e-passports. The EU Justice and Home Affairs Council decided to include fingerprints as a second mandatory identifier on passports in 2004.^11^ In addition, RFID can be used in e-ID cards in various countries. For example, in the United Kingdom, Prime Minister Tony Blair and his Labor Party convinced the nation to adopt biometrically-enhanced national identification cards (Ezovski and Watkins 2007). Tony Blair’s administration announced its will to implement RFID tag embedded national identification card in late 2004. China is another case where the e-ID card is used today. As a matter of fact, China is the country where e-ID card is widely and largely adopted today. The Beijing Olympics held in 2008 lit the fuse of adoption. The largest smart card project was implemented as a part of preparing the most prominent international sports event. In 2008, the Chinese government supplied 1.2 billion dollars of RFID readers and 2.25 billion dollars of RFID embedded smart cards to citizens. This made China the world’s largest market for RFID (Kovavisaruch and Suntharasaj 2007) (Table 3).

**Table 3:** RFID application in identification

Application	Paper	Year	Case	Country
e-passport	Meingast et al.	2007	US Visit Visa Waiver Program(VWP) focusing on why US government adopted RFID based e-passport	US
	Lorenc	2007	History of e-passport	US
	Ramos et al.	2009	Disadvantage of using e-passport from privacy perspective	US
	Kim	2008	Pros and cons of ROK government using RFID embedded e-passport	ROK
	Hossain	2014	How Australia adopt and implement e-passport	Australia
e-ID	Ezovski & Watkins	2007	e-ID used in United Kingdom	UK
	Kovavisaruch & Suntahrasaj	2007	Brief history of Chinese e-ID card	China

### Environmental applications

RFID technology can be widely applied in environmental applications. Adopting an RFID system in waste management is the most prominent way of using RFID to ensure efficient, eco-friendly waste management among lots of countries in the world. PAYT (Pay-As-You-Throw) program done by European Union (EU) is the pioneer of this field. PAYT is an RFID based waste pricing model that allows each individuals or each household to pay for the tag along with the total amount of waste they throw. Since each household and individual has a waste box in which RFID tags are embedded, the exact volume of waste can be calculated. In Europe this incentive based system has been proven to be a powerful policy tool for reducing the total amount of waste and for encouraging recycling (Schindler et al. 2012). Similar systems are broadly implemented in US (Ransford et al. 2012). In South Korea, the ministry of environment introduced it to industry and urged them to use an RFID based waste management system, especially in medical waste management. RFID technology is implemented in waste management in developed and developing countries, but the purpose of adoption is somewhat different from Europe to the US. India, the second-most populated country in the world, has adopted RFID technology to cope with the rapid increase of volume and types of waste (Infotech 2013). Similarly in 2010, what China faced were the World Expo and huge amounts of construction waste that comprised 30 % to 40 % of the total urban waste. Shanghai was chosen for a pilot project using an RFID based waste management system. All the waste dumping trucks had an embed RFID tag and volume of waste they carry was checked by the local government (Ruan and Hu 2011). Another interesting case of environmental application emerges from South Korea. The South Korean government operates U-Street Trees Systems through which the exact location and status of street trees can be monitored. Information about location and status of street trees are collected by an RFID tag that is attached to each tree is saved in a web information system, so trees can be managed effectively. Kim et al. (2006) claim that this web based information system could manage information remotely with an interactive system (Table 4).

**Table 4:** RFID applications in defense and security

Application	Paper	Year	Case	Country
Waste Management	Kang et al.	2012	Ministry of Environment in the ROK introduced RFID system in the medical waste management in 2006	ROK
	Infotech	2013	India adopted RFID technology in order to deal with rapid increase in volume and types of waste	India
	Ruan and Hu	2011	Shanghai city government started RFID using waste management to prepare Shanghai expo 2011	China
	WWICS	2008	How US government using RIFD in waste management	US
	Schindler et al.	2012	Cases of using RFID in waste management in EU member country	EU
	Ransford et al.	2012	Waste management operating system broadly used within US	US
Street tree Management	Kim et al.	2006	ROF government using RFID technology to manage street tree condition	ROK

### Transportation

Public transportation is another popular sector for RFID technology applications. RFID based electronic toll collection technology is one of the oldest and widespread RFID implementation (Ulatowski 2007). As soon as an RFID tag embedded car arrives at a toll booth, the RFID reader scans and reads the information that the RFID tag contains. The driver will pay debits according to the price that electronic reader suggests. In the US, electronic toll collection is thought as efficient and effective method that eliminates long lines of traffic at toll booth (Ulatowski 2007). RFID based toll collection is also adopted in criminal cases because it enables prosecutors to identify the exact location of the criminal’s car (Smith 2006). In South Korea, the Korean government has set credit card-linked electronic toll collection system called ‘Hypass’ especially for collecting transportation tolls on express ways. If an RFID tag is embedded on their cars, drivers can pass the tollbooth without stopping the car because RFID reader scan the data immediately and handle the whole payment process in about 5 s (Kim 2008). Hong Kong launched similar public transportation toll collection system in 1997 and the ‘Octopus Card’ is now internationally famous for its convenience. This system is able to handle 10 million transactions per day and includes all modes of public transport (Kovavisaruch and Suntharasaj 2007). South Korea has set credit card-linked electronic toll collection system called ‘Hypass’ especially for collecting transportation tolls on express ways. RFID technology is also implemented in railroad toll collection in India, where railroads are the most widely used form of public transportation. If an RFID tag is embedded on their cars, drivers can pass the tollbooth without stopping the car because RFID reader scan the data immediately and handle the whole payment process in about 5 s (Kim, 2008). In addition, RFID has been used as a critical technology to promote efficiency and transparency for public transportation system in developing countries. For instance, the Mexican government runs “Creating Traffic Knowledge in Mexico: Applying RFID to prevent vandalism” and one of the purposes of this innovative project is to develop a transportation information system to acquire more subtle data necessary for government decision making (Prado et al. 2010). Analogous to Mexican case, in Bangladesh where BRTA (Bangladesh Road Transport Authority) was started in 2003, the technology is operated mainly for control and supervision of the road transport systems (Hossain et al. 2009). RFID technology is also implemented in railroad toll collection in India, where railroads are the most widely used form of public transportation (Table 5).

**Table 5:** RFID application in transportation

Application	Paper	Year	Case	Country
Public Transportation	Ulatowski	2007	Electronic toll collection	US
	Kovavisaruch and Suntharasaj	2007	Well-known success smart card case in Hong Kong	Hong Kong
	Kovavisaruch and Suntharasaj	2007	Largest smart card transport system in the world	China
	Pransanth and Soman	2009	RFID based transportation system especially for railroad toll collection in India	India
	Kim	2008	Hypass case used in highway toll collection in ROK	ROK
Managing Road condition	Prado et al.	2013	How Mexican government using data collected from RFID system in decision making	Mexico
	Hossain et al.	2012	Monitor and control the road system	Bangladesh

### Healthcare and welfare

RFID enables hospitals to manage their equipment more easily and save expenses in public health areas^12^. The US government agencies like FDA have also already used RFID tag in monitoring drug industry^13^. Since American hospitals handle almost 4,000 medicines per day, medication errors can be easily occurred. With strong government support, public hospitals in Taiwan have actively adopted innovation of RFID (Kuo and Chen 2008)^14^. Even though it is not yet commercialized, an RFID identification system^15^ for the visually impaired people is being developed by engineers in Pakistan with the support of the Pakistani government (Murad et al. 2011) (Table 6).

**Table 6:** RFID application in healthcare and welfare

Application	Author	Year	Case	Country
Managing Public Hospital	Kuo and Chen	2008	Many hospitals are actively involved RFID system in managing hospital with the support of the government	Taiwan
Pharmacy	Wyld	2008	U.S. government and FDA recommended that pharmaceutical industries move to implement RFID tag to prevent counterfeit dugs by 2007	US
	Thuemmler et al.	2007		
	Romero and Lefebvre	2013		
	Skinar	2005	Florida state government imposed fine to drug suppliers when they did not adopt RFID tags	Florida State
Service for the impaired	Murad et al.	2011	RFID tag using service for visually impaired people was designed and implemented by Pakistan government	Pakistan
Infection Management	Nicholas	2008	Singapore government track visitors, patients and staff, to figure out who was the SARS virus bearer in 2003	Singapore
	Kuo et al.	2004	Taiwan hospital proceed RFID plans to track SARS patients	Taiwan

### Agriculture and livestock

RFID technology can be an effective tool for securing food safety and managing agriculture and livestock. Another major advantage to this system is that animal disease tracking can be realized through innovative technology like RFID (Hossain and Quaddus 2009). With the government support, researchers have developed the Navigation System for Appropriate Pesticide Use as a basic system for risk management in agriculture (Nanseki et al. 2005). RFID technology in agriculture was first introduced by the European Union (EU) in the late 1990s and shortly thereafter many countries, such as Australia, Japan and South Korea, adopted the innovation. Among those countries, the Australian government was the most passionate in implementing RFID^16^. For instance, all the livestock in Australia have RFID embedded tags on their bodies immediately after they are born; information that enables farmers to identify each entity and its health status is registered in National Livestock Identification System (NLIS). RFID technology in Japan has been also adopted in agriculture especially to secure food safety and agricultural risk management that can occur by abusing pesticides (Nanseki et al. 2005, Sugahara 2009). The Japanese government planned to make a food traceability system by 2010 as a part of the “e-Japan” plan (Chen et al. 2008). The United States is another case that applies mandatory RFID based identification system in managing livestock. According to RFID Gazette (2006), the USDA is pushing for RFID tagging of cattle to make tracing of disease patterns easier. With the formation of National Institute for Animal Agriculture (NIAA) in 2002, the plan for setting the National Animal Identification System was started. What the US government fulfilled through this program was “to be able to identify all animals and premises that have had contact with a foreign or domestic animal disease of concern within 48 h” (Wyld, 2005) because “the sooner animal health officials can identify infected and exposed animals and premises, the sooner they can contain the disease and stop its spread (USDA-APHIS 2005)” (Table 7).

**Table 7:** RFID application in Agriculture and Livestock

Application	Paper	Year	Case	Country
Agricultural risk management	Nanseki et al.	2005	Navigation system for Appropriate Pesticide Use was developed as a system for agricultural risk management	Japan
	Sugahara	2009		
Livestock Management and Disease Tracking	Trevarthen and Michael	2007	Cochrane Dairy Farm let their cows in herd have National Livestock Identification System	Australia
	Hossain	2009	How Australian farms adopt and implement RFID technology	Australia
	Wyld	2008	US began National Animal Identification System in 2002	US

## Public policy issues from RFID diffusion

RFID applications and diffusion generate complex policy and governance problems. We address public policy issues such as technological gap and uncertainty of expecting potential benefits and costs from a rapid and massive RFID diffusion. Uneasy governing issues in transparency, digital identification and power distribution are arising from inappropriate RFID applications. We discuss governance issues such as corruption, privacy problem, and digital monopoly and literacy in the following.

### Technological concerns

Technology is not still enough to satisfy all the elements that RFID is trying to perform various operational mechanisms. RFID technology deficiencies inevitably occur with the application of technology because there is niche space still left. For instance, RFID technology does not have a unified frequency standard yet. Since there are no internationally agreed upon frequencies for RFID operations, permitted scanner/reader powers also differ between countries. There are still significant differences between the frequencies from the EU and the USA (Hossain et al., 2009). In addition, Reichenhach (2008) pointed out the lack of storage capacity. In the EU, where RFID based waste management is common, there are technological barriers like a shortage of storage capacity^17^. Ema and Fujigaki (2011) draw implications from a child monitoring case done in Japan that being informed of children’s exact location cannot guarantee their actual safety, but RFID tags often lead to that cherished illusion. Vining (2005) warned about another possibility of niche space. According to his study about port security in the US, stealing goods without damaging RFID tag is possible because at ports, the container can be drilled into and contents can be removed. The RFID tag does not have to endure any damage through this whole process. In the US, as a response to continued pressure from various stakeholders, the US government even adopted the ‘Faraday cage’ for privacy protection^18^ (Table 8).

**Table 8:** Technological Issues

Issue	Paper	Year	Case	Country
Cryptography	Laurie	2007	Cryptography is not a perfect technique for protecting information saved in RFID tag so it can be easily attacked by the hackers	US
Standard	Hossain et al.	2009	No internationally agreed frequencies for RFID operations	
Storage Capacity	Reichenbach	2008	There is shortage of capacity for source-separated waste in household	Europe
NicheSpace	Ema and Fujigaki	2011	Informing children’s exact location cannot guarantee their actual safety	Japan
	Vining	2005	In port, the container can be drilled into and contents can be removed without disturbing the RFID tag	US
	Ezovski et al.	2007	Without any additional protections, the Faraday cage is not safe enough	US

### Uncertain cost-benefit effectiveness

RFID defenders emphasize that RFID technology can guarantee effectiveness and efficiency at a very cheap price. There is, however, substantial evidence to show RFID can generate unexpected costs^19^. In reality, the RFID tag is much more expensive than a barcode, which was very popular in identifying materials before the rise of RFID technology (Becker 2004). Purchasing RFID devices, hardware, and tags is not sufficient to drive system relevantly. To guarantee a better quality of service, the RFID system needs more additional things such as “circular process mechanism, the richness of consultant, project manager, programmers and plentiful project labors” (Kuo and Chen 2008). These elements for a better RFID performance may involve considerable costs. Kuo and Chen (2008) reported that RFID technology consumers and government should pay the extra hidden cost in the healthcare industry (Table 9).

**Table 9:** Cost-benefit effectiveness issues

Issue	Paper	Year	Case
Cost	Becker	2004	RFID tag is much more expensive than barcode
Hidden Cost	Kuo and Chen	2008	There is still hidden cost for highly qualified RFID system
Privacy	Jensen	2008	Active tag, which is more safe for securing privacy than passive tag is more expensive than passive tag

### Dubious transparency and corruption

RFID technology is expected to increase transparency and monitor corruption. However, RFID technology cannot ensure a high level of transparency than expected. As a matter of fact, RFID tags can be cloned and manipulated quite easily, and this kind of tag corruption can occur at every stage of RFID implementation. There are various examples to show an inappropriate use of RFID technology. For instance, Armknecht et al. (2010a, b) warned the possibility of tag corruption. Lee et al. (2012) pointed out reader corruption of the RFID technology. An existing security model mainly focuses on the possibility of tag corruption, but reader corruption can hurt consumers’ privacy as seriously as tag corruption can. Jules (2006) reported one of the tag corruption cases that observed in United States. One of the staff members who worked in a Dupyu store, an unscrupulous retailer, attached a cloned tag to counterfeit drugs. Avoine et al. (2010) argued that internet based databases can also be directly attacked and emphasized the possibility of reader corruption. There are also unethical behaviors to avoid RFID monitoring process. In the EU where an RFID based waste management system is aggressively implemented, some people disposed waste that came from their house at work places in order to avoid exact calculation through the RFID system. Not only this, Bilitewski (2008) reported that some conscienceless people are burning or transferring waste outside instead of throwing it into their RFID tag attached garbage can (Table 10).

**Table 10:** Transparency issues

Issue	Paper	Year	Case	Country
Tag Corruption	Armknecht et al.	2010	Tag corruption can be occurred at every stage of RFID implementation	
	Jules	2006	Dupyu staff attach cloned tags to counterfeit drugs	US
Reader Corruption	Lee et al.	2012	Although reader corruption can cause serious privacy attack, most of the scholars do not consider it as security problem.	
Cheating	Reichenbach	2008	Waste being disposed of at work places	EU
	Bilitewski	2008	Burning waste or transferring it to outside	EU

### Privacy issues

One of the most serious issues that RFID technology faces today is whether RFID technology is secure enough to protect privacy. Privacy is the most important concern RFID users have to deal with (Perakslis and Wolk 2005). RFID tag embedded chips often contain important personal information and usually this kind of private information can hurt one’s privacy seriously if leaked. To prevent leakage of private information, engineers developed cryptography, but there remains criticism^20^. The reason why these sorts of privacy concerns arise is because of the lack of security protection capacity of modern RFID technology. As we discussed above in the technological issues section, RFID technology today is not developed to secure perfect privacy. The technology itself has lots of deficiencies and people are smart enough to find niche spaces that can destroy the RFID security process. RFID itself can involve not only various hidden costs^21^ but also induces a serious privacy problem^22^. However, despite these possibilities of attacks on privacy, there are lots of stakeholders and scholars who advocate the potential benefits of RFID. They claim that tracking and profiling consumers is solely for implementing RFID chips more effectively. Eaward Rerisi, one of the producers of early implementation of RFID technology argued that, “An RFID reader can read the number on a tag, but without knowing what the number means, there is no way to access personal information. The idea that the tags can be read by just anybody—that’s pretty impossible” (Murray 2003) (Table 11).

**Table 11:** Privacy issues

Paper	Year	Category	Contents
Hwang et al.	2009	Cloning	The attacker can read the tag and then clone the tag by writing all the obtained data into other
		Eavesdropping	The attacker surreptitiously listens to all the communications between the reader and the tag
		Replay attack	The attacker repeats or delays the same message when valid data are transmitted
		Denial of Service	The attacker can send massive message to RFID system and attempt to crash the RFID system
		Forward Security	The attacker can compromise a tag and obtain its current relation date
		Tag tracing	The tag always broadcasts a fixed serial number to somewhere nearby the reader; therefore, the adversary can identify a fixed serial number of the tag from different locations or transaction records
		Individual data privacy	The hacker can know what items the consumer bought from the store or what books the consumer borrowed from the library
		Data forging	The attacker can modify the dates, items, and prices and then cause great loss if the tag can store extra data
Numann and Hogben	2008	Skimming	The attacker opens a clan-destine connection to the chip and gains access to the data
		Eavesdropping	The attacker intercepts the communication between the chip and an authorized reader
		Location tracking	The attacker generates person or card-specific movement profiles.

### Unequal power and digital literacy

Unequal distribution of RFID technology can generate unequal distribution of various resources such as information and digital literacy. Especially in developing countries, the combination of unbalanced power distribution between stakeholders and a low level of digital literacy can cause serious problems. Ketprom et al. (2007) emphasized that in developing countries like Thailand^23^, governments should provide education and training on how to use brand new technology to poor farmers whose digital literacy remains relatively low. But poor farmers in Thailand are not the only stakeholders who are suffering from a lack of digital literacy. In Bangladesh, where RFID toll collection is common, traffic policies have no interest in using RFID technology for managing public transportation systems. Rather, they prefer traditional ways of toll collecting to information based technology (Hossain et al., 2009). Prasanth et al. (2009) found that the lack of digital literacy among the Indian people hampered an effective process of railroad toll collection in India. Another problem developing countries face is an unbalanced power distribution due to lack of democratic value embedded governance. Chen et al. (2008) criticized the Taiwanese government because it monopolizes most of the information collected by RFID technology. As we already discussed above, when RFID tag scanned, information saved in RFID tag is scanned by reader and then transmitted to an internet based database. If that data were available to the public, individuals and industry could make more reasonable decisions by analyzing them. We find another unbalanced power distribution case in China’s waste management system. According to Ruan and Hu (2011), the Chinese government benefits most from the RFID system^24^ (Table 12).

**Table 12:** Power distribution and digital literacy issues

Issue	Paper	Year	Case	Country
Digital Literacy	Ketprom et al.	2007	RFID technology is widening the gap between poor and rich farmers	Thailand
	Hossain et al.	2009	Traffic policies are not interested in using RFID technology	Bangladesh
Power Distribution	Ruan and Hu	2011	Price of construction waste transportation is set by government, so other stakeholders have no choice	China
	Chen et al.	2008	Consumer can get only limited information	Taiwan

## Discussion and Conclusion

We found, relying on a systematic review from 111 RFID studies, six key areas of RFID applications. Specifically in the defense and security section, we addressed how military and airports/ports manage RFID systems to ensure security. We also found that RFID is effectively implemented in prison management and child protection programs. Numerous governments have introduced RFID identification tools such as e-passport and e-ID. RFID systems for waste management and street tree management are widely used from rich to poor countries. In healthcare and welfare delivery, RFID based smart cards have turned out to be very efficient. RFID is now being used to monitor counterfeit drugs. RFID has been applied to delivering service for the impaired and to trace infection. However, despite potential benefits from RFID applications, various unexpected problems arise. RFID can still involve technological deficiencies, especially in securing cryptography techniques, international standards of frequency, and storage capacity. RFID technology is not still enough to be efficient and effective in some areas (Becker 2004, Jensen et al. 2007). Tag and reader corruption can hurt transparency and security. Privacy issues are still the most serious issues that RFID faces today (Naumann and Hogben 2008). RFID itself can generate new unequal digital literacy and power distribution, especially in developing countries such as Thailand and Bangladesh. Even the most latest innovative technologies, like RFID, do not have perfect answers to securing efficiency, effectiveness, convenience, and transparency. Rather, RFID technology itself creates unexpected problems. It should be noted that democratic governance and trust is still important to technological innovation and policy issues arising from a rapid RFID diffusion.

Our systematic review is incomplete to discuss all of the RFID issues from technology, market and management, e-government, and legal aspects. Further research on RFID diffusion and impact include not only various theoretical issues of but also legal and managerial problems. For instance, both qualitative and quantitative research is required to explore what factors are critical to adopt and implement new RFID technology in terms of governance and digital literacy. Both micro and macro approaches with massive data are also required to identify how RFID improve not only organizational performance in government agencies and various industry sectors but also quality of our life.

## Footnotes

1 For example, after serious attack by Osama Bin Laden on 9/11, the American government decided to implement an RFID tag embedded e-passport and VISA waiver program. The US government asked their member countries to implement e-passport by late 2005 and soon US member countries like ROK and EU started to use e-passports. Currently, no one can enter to United States without an RFID tag based e-passport.

2 Especially in developing countries, governments usually adopt brand new IT technologies, but their low level of socio-economic infrastructures may prohibit the efficient operation of technology.

3 For instance, RFID applications may lack social virtues like trust, ethics, and democracy. It is essential to understanding how a rapid diffusion and massive applications of RFID generate conflict or harmony among human behaviors, digital literacy, institutional rules, and technology.

4 US Army and its allies could not only manage weapons and soldiers but also identify who was the enemy or not (Castro and Wamba, 2007). This whole project of developing an RFID based identifying system was known as IFF (Identify Friend of Foe).

5 See more various examples of RFID applications at the website (https://epic.org/privacy/rfid/)

6 In 2004, the US Army adopted RFID during the Iraq war to track Iraq troops. Not only these, the US Army piloted 4 projects using RFID; identifying material locations, weapons deteriorations, hazardous material tracking, and asset tracking (Anon 2002).

7 The New York City government also started an RFID e-seal pilot project in the New Jersey Port. Once RFID read and scan the tag, it can identify the contents of the container box. Also, the port of Tacoma and Seattle planned to adopt E-Seals, made of metal bolts with embedded RFID devices to ensure its security (Konsynski and Smith 2003).

8 Calpatria prison, located in Los Angeles, adopted a prisoner monitoring system using RFID chips in 2000 as the very first pilot using RFID in prison management in United States (Kim 2008). According to regulations of Calpatria prison, all the prison inmates were issued bracelets in which RFID chips were embedded. Since the pilot project at the Calpatria prison was successful, the local government let other prisons in Los Angeles adopt the innovative bracelet. The LA County prison started to use a brand new bracelet in response to the state government’s order; it is reported that through its adoption the prison could increase efficiency and effectiveness and decrease crimes occurred between inmates simultaneously (Nicholas 2008).

9 For instance, city governments in the Gifu and Osaka prefecture provided RFID tags that can be attached in students’ schoolbags to public elementary schools (Ema and Fujigaki, 2011). Similarly, in Haewoondae beach, one of the most famous vacation spots in South Korea, Busan Metropolitan City provides for parents RFID embedded bracelet that enables tracking exact location of their child by a smart phone (http://news.naver.com/main/read.nhn?mode=LSD&mid=sec&sid1=102&oid=001&aid=0003353674).

10 This project began in 2002, but it took 3 years to fully implement for all 16 US passports (Meingast et al. 2007). The appearance of the e-passport is very similar to old passports, but woven into the paper of the passport, there is RFID tag that information about owner of the passport is included. Information about nationality, sex, age, and so on is scanned, as airport staff members scan the passport through RFID reader (Lorenc 2007). The US government did not stop at this point and adopted VISA Waiver Program. By 2005, the US member countries had to adopt RFID based e-passports and VISA Waiver Programs in order for their citizens to enter the United States because without e-passports, passengers could not be accepted at American points of entry. Today, the e-passport includes not only individual data but biological data, such as fingerprints (ICAO TAG MRTD/TWF 2004).

11 E-Government News, “EU Asks US for Time to Issue Biometric Passports”. iDABC European e-Government Services, 1 April, 2005.

12 For instance, Wicks et al. (2006) reported that this RFID based hospital management system is very effective in reducing management costs because embedded RFID tags can track lost or hidden expensive equipment. Miller (1999) pointed out that the potential of RFID technology can be expanded in tracking the location of patients and controlling the drugs. Also, Chowdhury and Khosla (2007) argued that RFID technology can be effectively used not only in hospital equipment management but also in patient management.

13 According to statistics published by the Institute of Medicine (IOM), about 44,000 to 98,000 people die in the USA per year because of improper drug administration (Kohn et al. 2000). To rectify this phenomenon, in 2004 the US government and US FDA recommended pharmaceutical industries to implement RFID tags to prevent the production of counterfeit drugs (Wyld, 2005). The Florida state government added legal regulations to this recommendation in 2006. If a pharmaceutical industry located in Florida does not attach RFID embedded tags on its products, it has to face substantial financial penalties (Skinar 2005).

14 For instance, RFID technology saved Singapore and Taiwan from SARS attack around 2003. Two public hospitals in Singapore in 2003 adopted RFID technology to track staff, patients, and visitors in order to trace people who carried the SARS virus. The RFID information database saved all the data collected from each individual’s RFID tag for 21 days, which was thought to be long enough for expression of SARS virus (Nicholas 2008). A similar process was done in Taiwan too. During the SARS period, five hospitals, including Taipei Veteran’s General Hospital, implemented RFID tags to track patients who had possibility contracted the SARS virus (Kuo et al. 2004) with strong government support.

15 One peculiar characteristic of this system as compared to other systems is that visually impaired people are both the reader carrier and the service beneficiaries, simultaneously. Generally in government service delivery using RFID technology, the service provider usually carries RFID tag readers and service beneficiaries usually act more passive roles by attaching RFID tags. But in this Pakistani case, the service beneficiaries can identify objects around them by operating RFID tag reader they have.

16 The Australian government passed legislation on mandatory use of RFID tags in the livestock industry, so Hossain and Quaddus (2014) categorized Australian case as a very rare and special adoption case. According to Trevarthen and Michael (2007)’s case study, one of the Australian farms where the RFID tag is implemented, farmers not only track the exact location of the cows but also check the condition, identify cows and even feed the new born cows automatically by using an RFID system.

17 Usually people throw waste in various places. They may throw it away at their houses or at work places, like an office. Since the RFID tag is only attached to a garbage can in house, it is impossible to track all types of waste throwing behaviors. Inevitably, this shortage of storage capacity leads to selective waste collection monitoring.

18 The faraday cage is an object in metal; proponents of this device argue that faraday cage can prevent hacker’s attack because electronic devices are prevented from passing through the object (Ezovski et al. 2007). But speculation about stability of this technology still remains. Lorenc (2007) reported that if there is no additional technology, the faraday cage cannot preserve sensitive security.

19 As a matter of fact, there are two kinds of RFID tags, the passive tag and the active tag. These two tags provide owners with different benefits and liabilities. Active tags are implemented by a power source, such as small battery. Active tags are more efficient and safe in protecting privacy than passive tags, so sensitive organizations like military prefer active tags to passive tags. But ordinary consumers have less accessibility to active tags because active tags are much more expensive than passive ones (Jensen et al. 2007).

20 According to Laurie (2007), even without physically losing an RFID tag, private information can be stolen because what’s inside RFID tags can be skimmed quite easily. If we can build a device that enables us to transmit an arbitrary number, invading the internet based database is theoretically possible.

21 For instance, Wal-Mart Stores Inc. and Procter & Gamble Co. in autumn 2003 planned a very interesting experiment to check the potential deficiencies of RFID technology. Customers of a Wal-Mart store in Broken Arrow, Oklahoma were secretly tracked through RFID tags while purchasing lipstick that Procter & Gamble Co. made (Barut et al. 2006).

22 For instance, Hwang et al. (2009) and Numann & Hogben (2009) categorized various kinds of privacy attack cases that can possibly occur. According Hwang et al. (2009), technological deficiency enables hackers to engage in cloning, eavesdropping, replay attack, denial of service, forward security, tag tracing, individual data privacy, and data forging. Specifically, the hacker can read the tag and then clone the tag (cloning), surreptitiously listen to all the communications between and the tag (eavesdropping), repeat or delay the message (replay attack), send large amount of message to break down RFID system (denial of service), compromise a tag (forward security), trace the exact location of the tag (tag tracing), find out shopping trend of the consumer (individual data privacy), and modify information saved on an RFID tag (Data forging). In addition, Numann and Hogben (2008) categorized the privacy attacking cases more briefly. According to their research, the hacker can attack RFID tag in some ways. First, the attacker can open a connection to the chip and can steal the data inside (skimming). Second, the attacker can intercept the communication between tag and reader (eavesdropping). Last, the attacker can track the exact location of the tag or the person.

23 In Thailand, most of the farms are trying to adopt an RFID system in farm management, but RFID technology is widening the gap between poor and rich farmers. Poor farmers are usually less educated people who have hardly had any experience using digital technology like RFID. On the other hand, well-educated wealthy farmers face low entry barriers and easily adopt the technology. Rich farmers armed with innovative technology not only make enormous fortunes by increasing efficiency but also by replacing poor labors with RFID embedded devices. According to the Thailand Ministry of Labor (2006), most farm labors are afraid of being replaced. Unfortunately, this phenomenon eventually correlates to a serious gap between rich and poor.

24 The government has invested a huge amount of money to buy necessary devices such as readers, tags, hardware, and so on, but in the long run cost of management will decrease. On the other hand, the waste industry could carry a very heavy debt. The waste contractors have to deal with expensive RFID tag rental and as well as the cost of construction simultaneously. Although this situation is totally unfavorable for them, the waste management industry cannot resist to this policy because the Chinese government is the entity which has made the use of RFID policy and set prices. The industry has no other choice but consent.
